# Dandelion extract relaxes mouse airway smooth muscle by blocking VDLCC and NSCC channels

**DOI:** 10.1186/s13578-020-00470-8

**Published:** 2020-10-28

**Authors:** Ping Zhao, Jia Liu, Qian Ming, Di Tian, Jingwen He, Ziwei Yang, Jinhua Shen, Qing-Hua Liu, Xinzhou Yang

**Affiliations:** 1 Institute for Medical Biology & Hubei Provincial Key Laboratory for Protection and Application of Special Plants in the Wuling Area of China, College of Life Sciences, South-Central University for Nationalities, Wuhan, 430074 China; 2 Hubei Medical Biology International Science and Technology Cooperation Base, Wuhan, 430074 China; 3 School of Pharmaceutical Sciences, South-Central University for Nationalities, 182 Min-Zu Road, Wuhan, 430074 China; 4 National Demonstration Center for Experimental Ethnopharmacology Education, South-Central University for Nationalities, Wuhan, 430074 China

## Abstract

**Background:**

Asthma is one of the main intractable diseases recognized by the international medical community. The current widely used bronchodilators for asthma—β2-adrenal receptor agonists—have limited therapeutic effects, necessitating the development of novel antiasthma drugs with increased efficacy and fewer adverse effects. In this study, we investigated the relaxant effects and underlying mechanism of an ethyl acetate extract from dandelion (EAED) on mouse airway smooth muscle.

**Methods:**

The effects of EAED on agonist-induced precontraction in mouse airway smooth muscle were evaluated with force measurement. Mouse lung slices were used to study the effects of EAED on bronchial smooth muscle. The intracellular Ca^2+^ concentration was measured using a calcium imaging system. L-type voltage-dependent calcium channel (VDLCC) and non-selective cationic channel (NSCC) currents were measured by patch-clamp. The lung functions of healthy and asthmatic mouse groups were assessed via the forced oscillation technique.

**Results:**

EAED inhibits acetylcholine-induced sustained contractions of whole airway smooth muscle by inhibiting VDLCCs, NSCCs, and some unknown channels, reduces the agonist-induced increase in the cytosolic free Ca^2+^ concentration in airway smooth muscle cells, blocks VDLCC and NSCC currents, and relieves the respiratory resistance of healthy and asthmatic mice.

**Conclusions:**

EAED may have potential beneficial effects on mitigating asthma attacks.

## Background

Asthma is a major chronic respiratory disease, threatening the health of hundreds of millions of people around the world [1], and has become a severe global public health problem [2]. Airway inflammation, airway hyperresponsiveness, and airway remodeling are important pathophysiological characteristics of asthma. Airway smooth muscle (ASM) is the key tissue regulating airway resistance, hyperreactivity, and contraction, the major features of asthma [3]. ASM cells (ASMCs) are an important cell type in ASM and excessive contraction of ASMCs leads to the development of asthma symptoms by narrowing the airway lumen and limiting gas exchange [3]. ASM contraction induced by agonists (*i.e.* acetylcholine, 5-hydroxytryptamine) usually relies on an increase in [Ca^2+^]_i_ and on Ca^2+^ oscillations. These oscillations are caused by the release of Ca^2+^ from the intracellular calcium pool and the influx of Ca^2+^ from the extracellular space [4].

The first-line treatment for asthma is still a combination of β_2_ adrenergic receptor agonists and glucocorticoids. However, this therapeutic strategy can have severe adverse effects, such as headache, tremors, palpitations, and heart failure [5–7]. Thus, in this study, we attempted to develop a safe and effective plant-based drug to inhibit ASM contraction.

Dandelion is a perennial herbaceous plant with the scientific name *Taraxacum mongolicum* Hand. -Mazz. (TMHM). Its main chemical components are taraxasterol, choline, organic acid, inulin, and other healthy nutrients [8]. It is thus recognized as a nutritious wild vegetable. In addition, it has many pharmacological effects. Modern pharmacological studies show that the properties of dandelion include antibacterial [9, 10], antiviral [11], anticancer [12–16], antioxidant [17], anti-inflammatory [18–20], and antiallergic functions. In terms of the alleviation of airway inflammation, a distinctive feature of asthma, it has been reported that the organic acid components of TMHM can improve lipopolysaccharide-induced histopathological damage to tracheal tissues [21] and reduce lipopolysaccharide-induced inflammation in normal human bronchial epithelial cells [22], which could be beneficial for the treatment of acute tracheobronchitis. Taraxasterol was also found to be effective in improving ovalbumin-induced allergic asthma in mice [23]. Abundant literature also concerns the potential efficacy of dandelion in mice. However, no studies have examined whether a specific component from dandelion has the potential to inhibit mouse ASM contraction.

In this study, we found that EAED exerted inhibitory effects on mouse ASM precontraction and investigated the underlying mechanism.

## Methods

### Dandelion extraction

Dandelion was purchased from Beijing TongrenTang (Wuhan, China). The air-dried dandelion (0.5 kg) was milled into powder and soaked in 80% ethanol (5L) for 3 days. Then the crude ethanol extract was obtained by filtration and rotary evaporation. The ethyl acetate extract of dandelion was obtained by phase separation extraction. The dried ethyl acetate extract of dandelion was dissolved in 3% DMSO for the experiments.

### Reagents

Nifedipine, acetylcholine chloride (ACH), and pyrazole3 (Pyr3) were purchased from Sigma Chemical Co. (St. Louis, MO, USA); Fura-2 AM were purchased from Invitrogen (Eugene, OR, USA). Other chemicals were purchased from Sinopharm Chemical Reagent Co. (Shanghai, China).

### Animals studies

Six-weeks-old male BALB/c mice were purchased from the Hubei Provincial Center for Disease Control and Prevention (Wuhan, China) and were housed in a specific pathogen free (SPF) grade animal facility. All animal experiments were performed in accordance with the requirements of the Institutional Animal Ethics Committee of the South-Central University for Nationalities. The license number is 2016-SCUEC-AEC-0030. Asthmatic mice were prepared as described previously [24]. And, the mice were sacrificed with an intraperitoneal injection of sodium pentobarbital (250 mg/kg, purity ≥ 98%; Sigma) before or after performing each experiment.

### Contraction measurement of tracheal and bronchial ASM

Mouse ASM tension was measured as previously described [25]. Briefly, mouse TRs were clipped clean, cut about 1 cm and hung on the triangular hook in a 6 mL PSS bubbled with 95% O_2_ and 5% CO_2_ at 37 ℃. A 300 mg preload is set. TRs was equilibrated for 1 h and then prestimulated with 100 μM ACH or 80 mM KCl for 20 min. After resting for another 20 min, experiments were performed.

ASM force measurements in mouse lung slices were performed as previously described. In brief, the lung slices were cut and placed in a chamber perfused using Hanks’ balanced salt solution (HBSS). The LSM 700 laser confocal microscope and Zen 2010 software (Carl Zeiss, Göttingen, Germany) were performed to measure the cross-sectional areas of the bronchial lumen. Each part of the experiment was independently repeated for more than six times (i.e., more than six random mice).

### Measurement of plasma calcium concentration in ASMCs

Mouse acute detached ASMCs suspension was diluted to an appropriate density and was treated with poly-d-lysine. A specially designed cell bath of the passed slides is placed in an inverted microscope connected to the calcium imaging system. Poly-d-lysine could help cells adhere to the glass slide. Then cells were dyed with 2.5 μM Fura-2 AM. After 20 min of staining, PSS physiological saline solution was perfusion for 5 min to wash away the excess fura-2. With calcium imaging TILL imaging system: 340 and 380 fluorescence images of the cell area, Ratio (340/380) can be used to reflect the intracellular calcium concentration. Each part of the experiment was based on more than 30 ASMCs (i.e., more than six random mice).

### Patch

VDLCCs and NSCCs currents induced by ACH were recorded using EPC-10 patch clamp amplifier (HEKA, Lambrecht, Germany) and utilizing whole-cell recording mode. VDLCCs current was stimulated by step voltage from − 70 mV to + 40 mV. NSCCs current was recorded with nifedipine, niflumic acid and TEA in the solution in advance, under the condition of the ramp, voltage from − 80 mV to + 60 mV in 500 ms. Each part of the experiment was independently repeated for more than six times (i.e., more than six random ASMCs/mice).

### Pulmonary function measurement

Lung function of groups of healthy or asthmatic mice were measured using forced oscillation technique (FOT). Mice were weighed and anesthetized with an injection of sodium pentobarbital (10 mg/kg, ip). After complete anesthesia, the mice were intubated and placed in a flow-type body plethysmograph and connected via the endotracheal cannula to a flexiVent system (SCIREQ Inc., Montreal, Canada). Lung function was assessed subsequently by FOT at baseline and following multiple concentrations of aerosolized ACH (3.125–50 mg/mL) dissolved with vehicle or EAED. Respiratory system resistance (Rrs) were calculated in the flexiVent software to reflect the degree of airway hyperresponsiveness. Each part of the group experiment was independently repeated for more than six times (i.e., more than six random mice).

### Data analysis

The results are expressed as mean ± SEM. Comparisons between two groups were performed with Student’s t-test using Origin 9.0 software (OriginLab, Northampton, USA). Differences with p < 0.05 were considered significant.

## Results

### EAED inhibits tracheal ring contraction

**Fig. 1:**
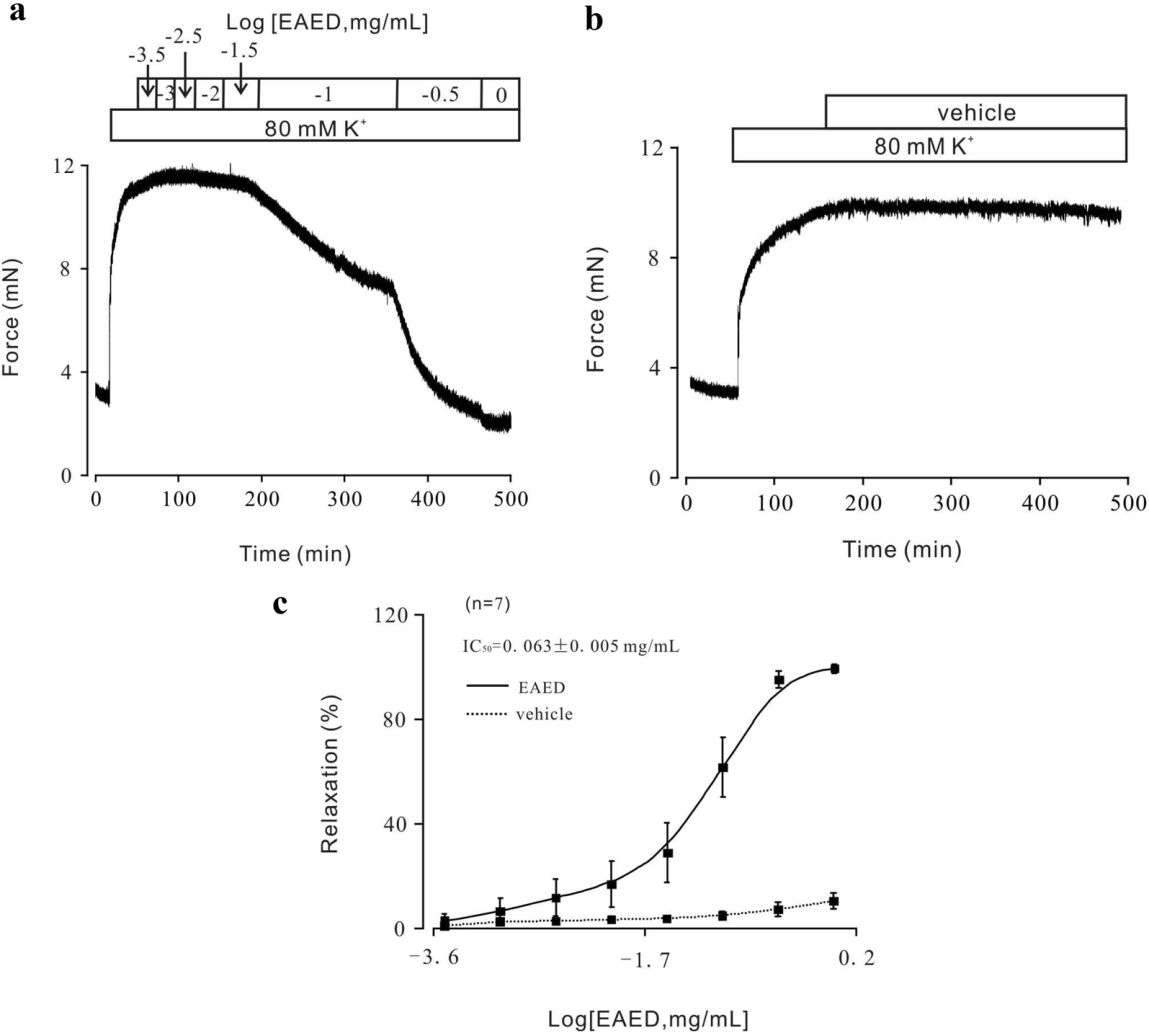
EAED inhibited high K^+^-induced tracheal ring contraction.** a** K+ (80 mM) induced a sustained contraction in mouse TR, which was blocked by EAED in a concentration-dependent manner. The dose-inhibition curve is presented.** b** Similar experiments were performed with vehicle (PSS containing 3% DMSO) as control.** c** The dose-inhibition curve is presented. The IC50 of EAED was 0.063 ± 0.005 mg/mL. The data were obtained from 7 TRs

We first studied the effects of EAED on tracheal ring (TR) contraction. TRs were precontracted with 80 mM KCl and EAED was added when the contraction reached a plateau. The contraction was inhibited in a dose-dependent manner (Fig. 1a). As a comparison, vehicle (PSS containing 3% DMSO), which was used to dissolve the EAED, was added at the same doses when the contraction stabilized (Fig. 1b) and no relaxation was detected. This suggests that EAED indeed relaxes ASM. The half-maximal inhibitory concentration (IC_50_) of EAED was 0.063 ± 0.005 mg/mL (Fig. 1c). We also found that the contraction induced by 80 mM KCl was almost completely inhibited at an EAED concentration of 1 mg/mL. These results were obtained from 7 TRs from 7 mice.

**Fig. 2:**
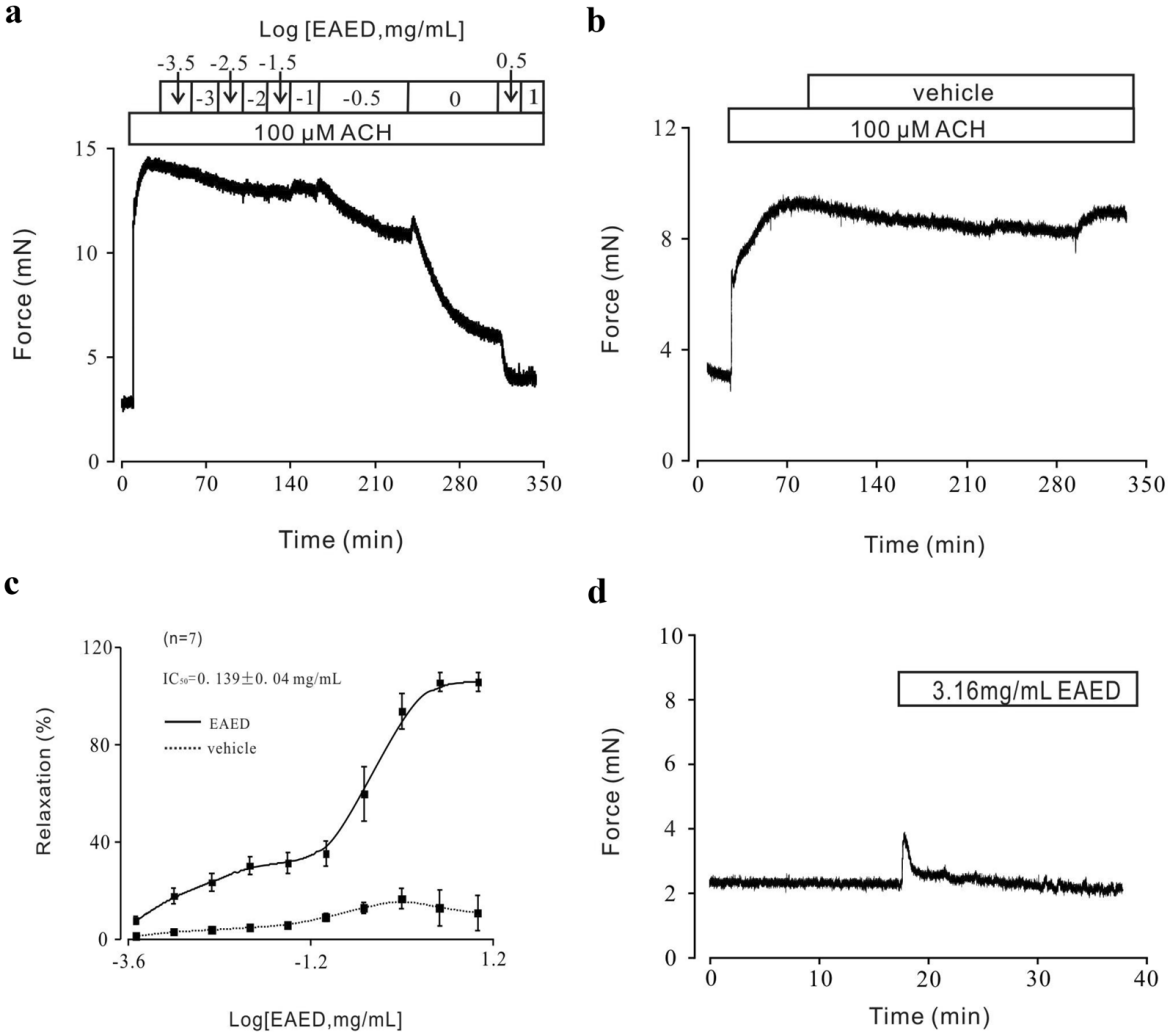
Contraction induced by ACH was inhibited by cumulative addition of EAED

Similarly, EAED was added after the contraction arising from 100 µM ACh) peaked, which induced a gradual but clear inhibition of the precontracted TRs (Fig. 2a). In addition, vehicle control (PSS containing 3% DMSO) was added at the same doses under steady contraction conditions (Fig. 2b), which again exerted no relaxant effects. Analysis of the dose-relaxation relationships determined an IC_50_ of EAED of 0.139 ± 0.04 mg/mL (Fig. 2c). The EAED concentration inducing maximum relaxation was 3.16 mg/mL. These experiments indicated that EAED could block high K^+^- and ACh-induced TR precontraction. In addition, the addition of 3.16 mg/mL EAED without pretreatment with any agonist resulted in a small immediate contraction and a subsequent return to baseline (Fig. 2d), which indicated that EAED had no effect on the TRs in the resting state.

### EAED blocks bronchial smooth muscle contraction

**Fig. 3:**
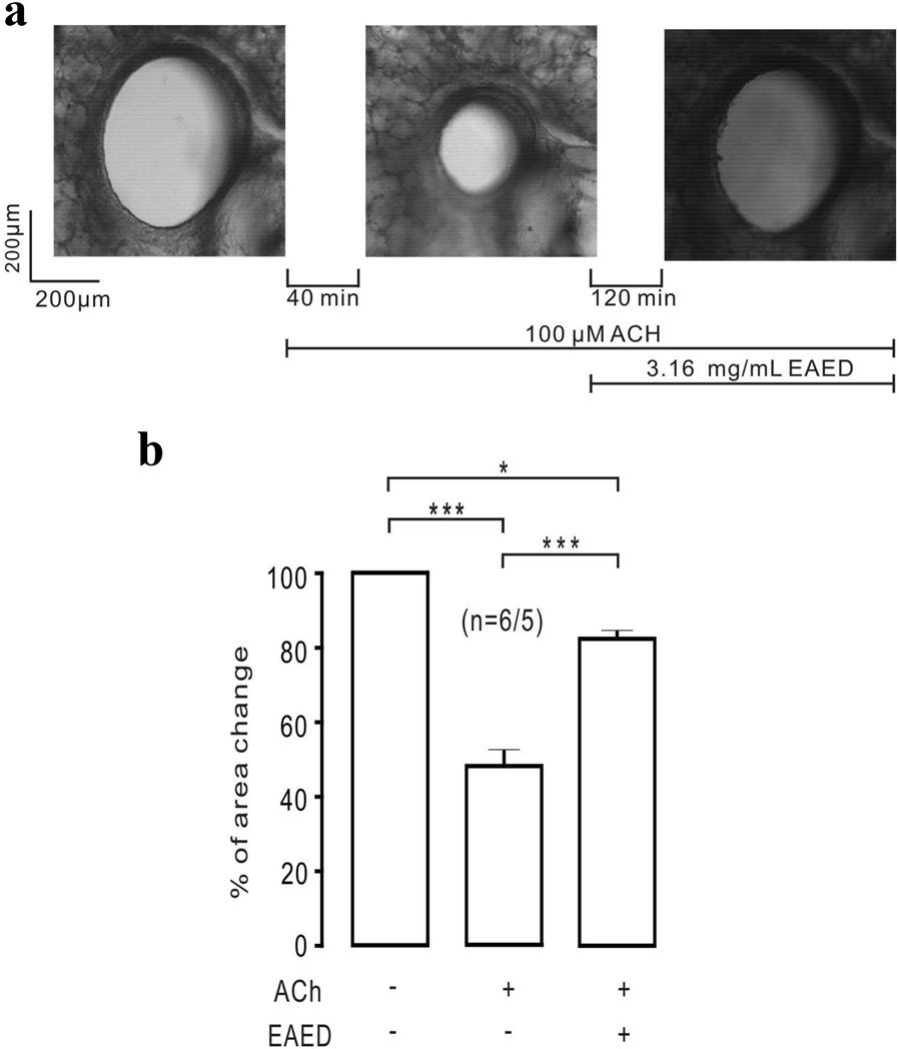
EAED inhibits contraction in lung slices

To investigate whether EAED has a similar relaxant effect on mouse bronchial smooth muscle, the effects of EAED on lung slices were examined. Treatment with 100 µM ACh decreased the tracheal cavity area; the addition of EAED restored the lumen area (Fig. 3a). A summary of the data from 6 lung slices from 5 mice is shown in Fig. 3b. After the addition of 100 µM ACh for 40 min, the area of the lumen reduced to approximately 48%; subsequent application of 3.16 mg/mL EAED for 120 min further decreased the area by about 82% reduction compared with the initial value. These results suggested that EAED may also inhibit the contraction of bronchial smooth muscle.

### EAED exerts diastolic effects by inhibiting L-type Ca2+, TRPC3, and/or STIM/Orai channels

**Fig. 4:**
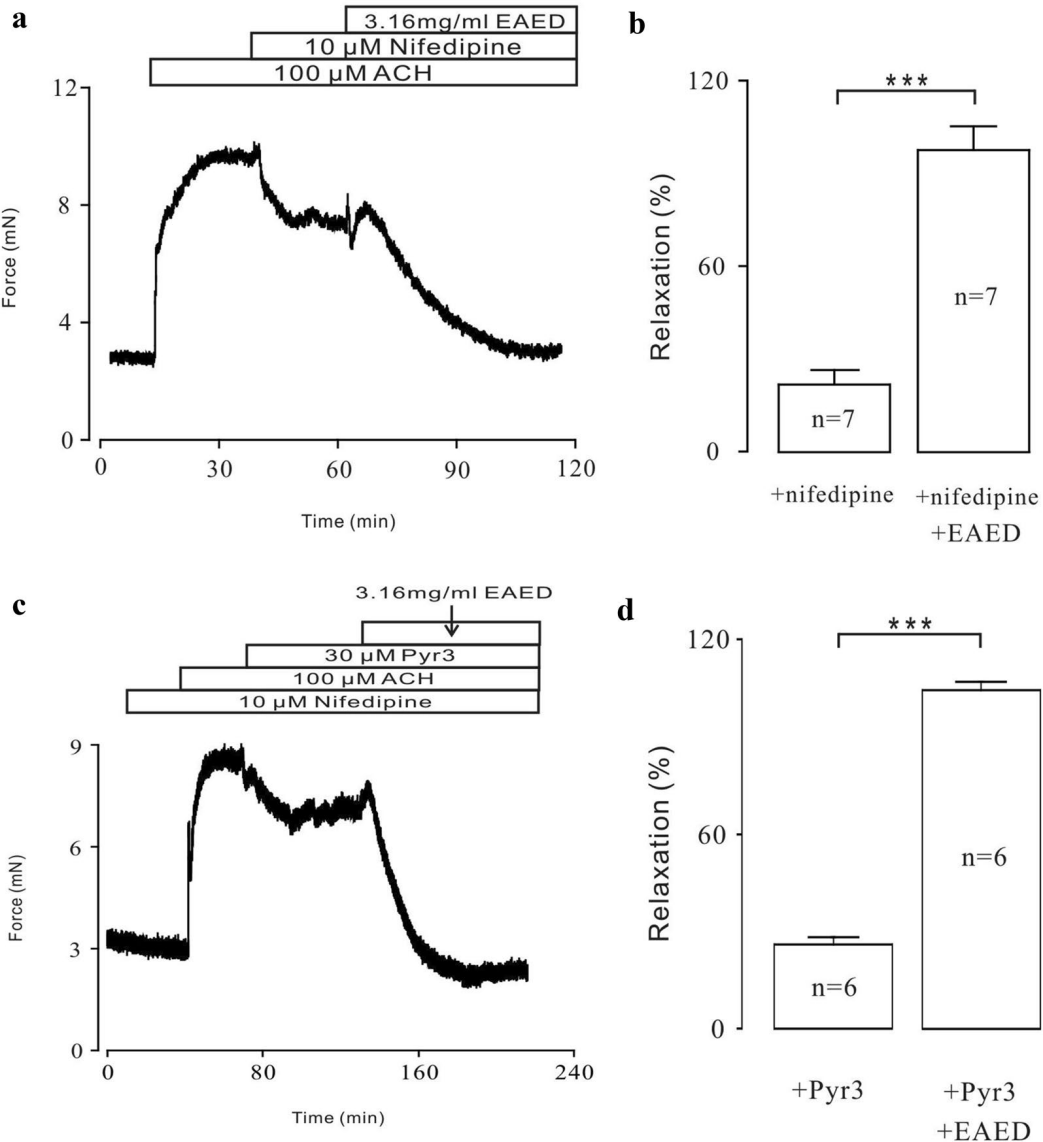
Nifedipine, Pyr3 partially inhibits ACH-induced contraction, respectively

To investigate the mechanism of the EAED inhibition of ACh-induced contraction, 10 µM nifedipine, a selective blocker of voltage-dependent calcium channels (VDCCs), was added after contraction was induced by ACh (Fig. 4a). The drug partially blocked the contractions, giving a relaxation value of about 18%. The remaining contractions were further blocked by EAED, with a relaxation of about 95% compared with baseline (Fig. 4b). These data were obtained from 7 TRs of 7 mice.

Next, we investigated the nifedipine-resistant components of EAED-induced relaxation. Hence, TRs were incubated with 10 µM nifedipine for 15 min and ACh was then added. The effect of Pyr3 was observed. The overall results from 6 TRs of 6 mice showed that Pyr3 induced partial relaxation (about 25%; Fig. 4c), with the remaining contractions completely blocked by EAED (almost 100%; Fig. 4d).

### EAED inhibits Ca2+ influx induced by high K+ and additional Ca2+ release induced by ACh

**Fig. 5:**
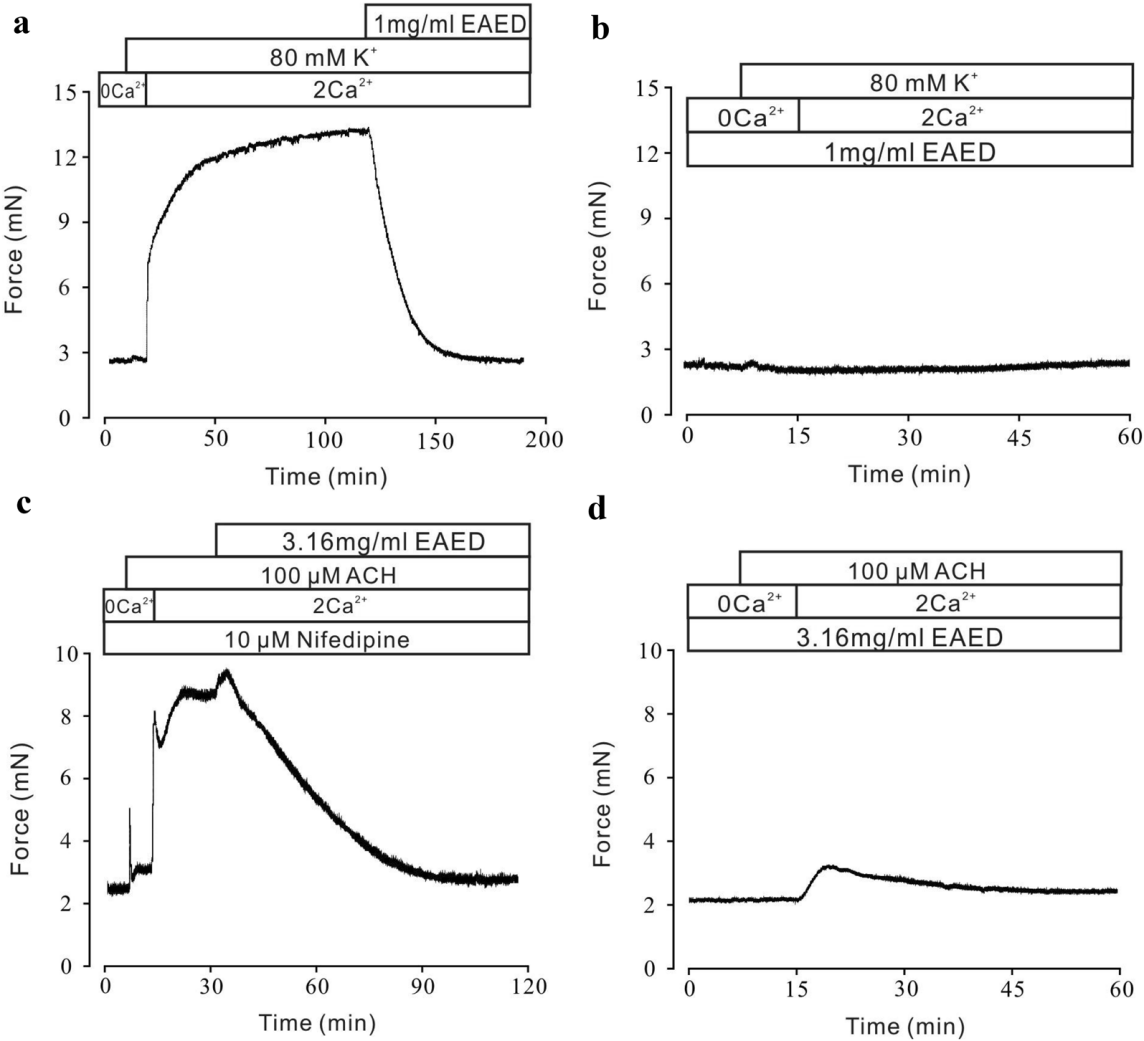
EAED blocks high K^+^-evoked Ca^2+^ influx and ACH-elicited Ca^2+^ influx and Ca^2+^ release

To further confirm the relationship between these channels and relaxation, a calcium-free and physiological calcium conversion experiment was designed. As shown in Fig. 5a, when the TR was at 0 Ca^2+^, high K^+^ still activated the L-type voltage-dependent calcium channel (VDLCC) without increasing the intracellular Ca^2+^ concentration. Thus, it could not cause TR contraction. When the extracellular [Ca^2+^]_i_ was returned to 2 mM, the extracellular Ca^2+^ flowed rapidly, the intracellular [Ca^2+^]_i_ increased, and the TR constricted. This contraction was inhibited by 1 mg/mL EAED. Furthermore, incubation with EAED almost completely abolished the contraction induced by 2 mM Ca^2+^ (Fig. 5b). From these results, it can be concluded that EAED relaxation of precontracted tracheal smooth muscle induced by high K^+^ was mediated by inhibition of VDLCCs and Ca^2+^ influx.

ACh can activate both VDLCCs and non-selective cationic channels (NSCCs), which leads to extracellular Ca^2+^ influx, release of Ca^2+^ from the sarcoplasmic reticulum into the cytoplasm, increased Ca^2+^ concentration, and ultimately contraction of tracheal smooth muscle. ACh was added under calcium-free conditions. Because there was no Ca^2+^ outside the cell, it caused a transient release of Ca^2+^ from the sarcoplasmic reticulum, leading to a transient contraction. When the extracellular [Ca^2+^]_i_ was restored to 2 mM, the Ca^2+^ in cytoplasm was increased by both the Ca^2+^ from the sarcoplasmic reticulum and the increase in extracellular Ca^2+^ (Fig. 5c). Thus, the trachea showed a continuous and stable contraction. This contraction was inhibited by 3.16 mg/mL EAED. Moreover, under Ca^2+^-free conditions (0 Ca^2+^ and 0.5 mM EGTA) in the presence of EAED, ACh did not induce a transient contraction. With the addition of 2 mM Ca^2+^, only a very weak contraction occurred, which gradually returned to baseline (Fig. 5d). These results indicated that EAED-induced relaxation was exerted through inhibition of the ACh-elicited Ca^2+^ influx and Ca^2+^ release.

### EAED inhibits Ca^2+^ elevation in single ASMCs

**Fig. 6:**
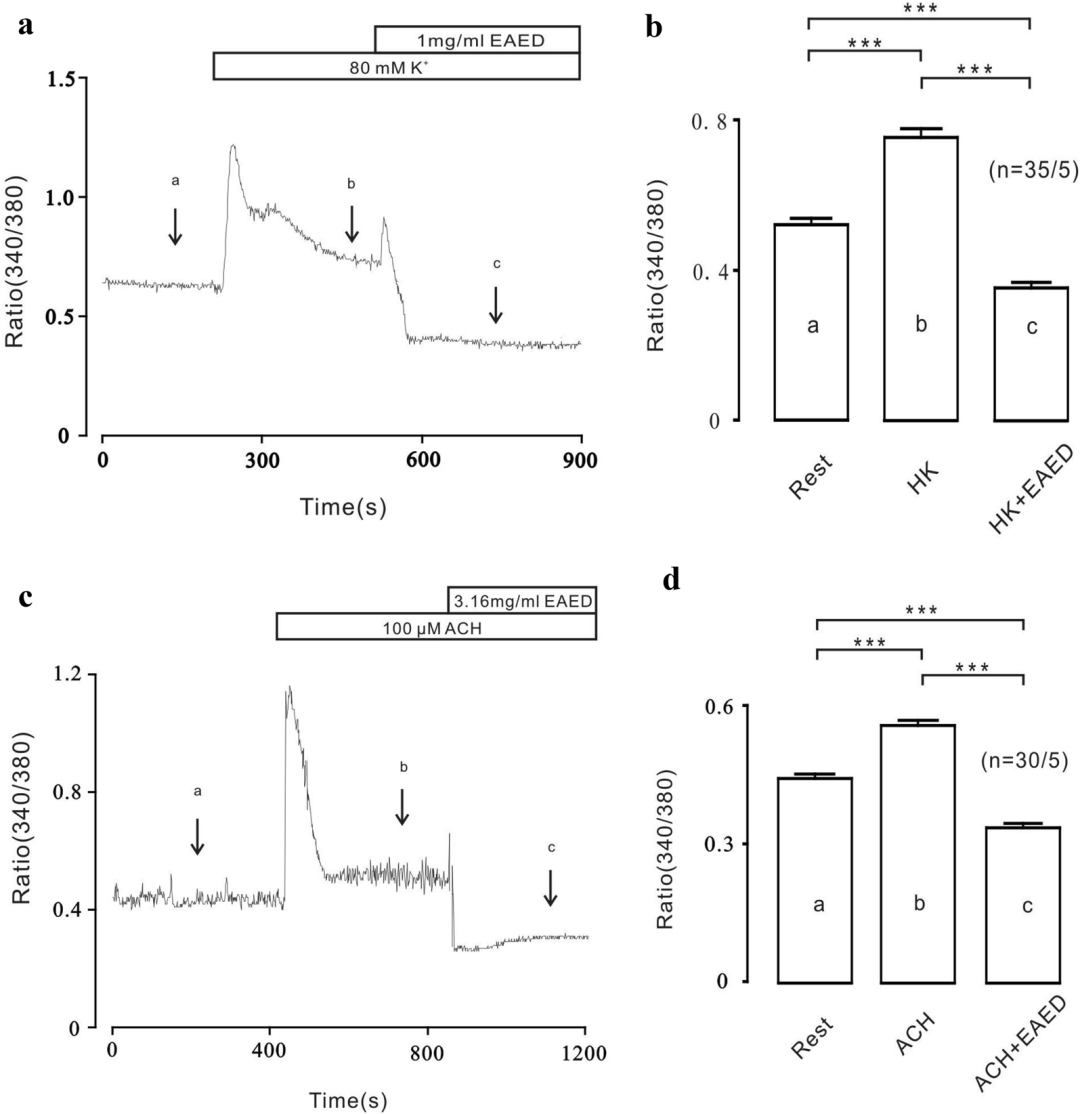
EAED inhibits high K^+^ and ACH-induced Ca^2+^ increases in single tracheal smooth muscle cells

Next, the effects of EAED on intracellular Ca^2+^ in single ASMCs were examined by use of the TILL calcium imaging system. High K^+^− (Fig. 6a) and ACh- (Fig. 6c) induced increases in intracellular Ca^2+^ were inhibited by 1 mg/mL or 3.16 mg/mL EAED. The 340/380 ratio at the sites indicated by a, b, and c were obtained and a summary of the results from 30–35 cells of 5 mice are shown (Fig. 6b and d). After the addition of high K^+^, the 340/380 ratio increased from 0.51 ± 0.01 at point a to 0.75 ± 0.02 at point b, before reducing to 0.35 ± 0.01 at point c with the subsequent addition of 1 mg/mL EAED. Similar results were found with the ACh-stimulated increase in [Ca^2+^]_i_, where the 340/380 ratio increased from 0.44 ± 0.01 at point a to 0.55 ± 0.01 at point b, before reducing to 0.33 ± 0.01 at point c with the subsequent addition of 3.16 mg/mL EAED. These results suggest that the [Ca^2+^]_i_ decreases were due to inhibition of the above Ca^2+^-permeant ion channels by EAED.

### EAED effectively blocks VDLCC and NSCC currents

**Fig. 7:**
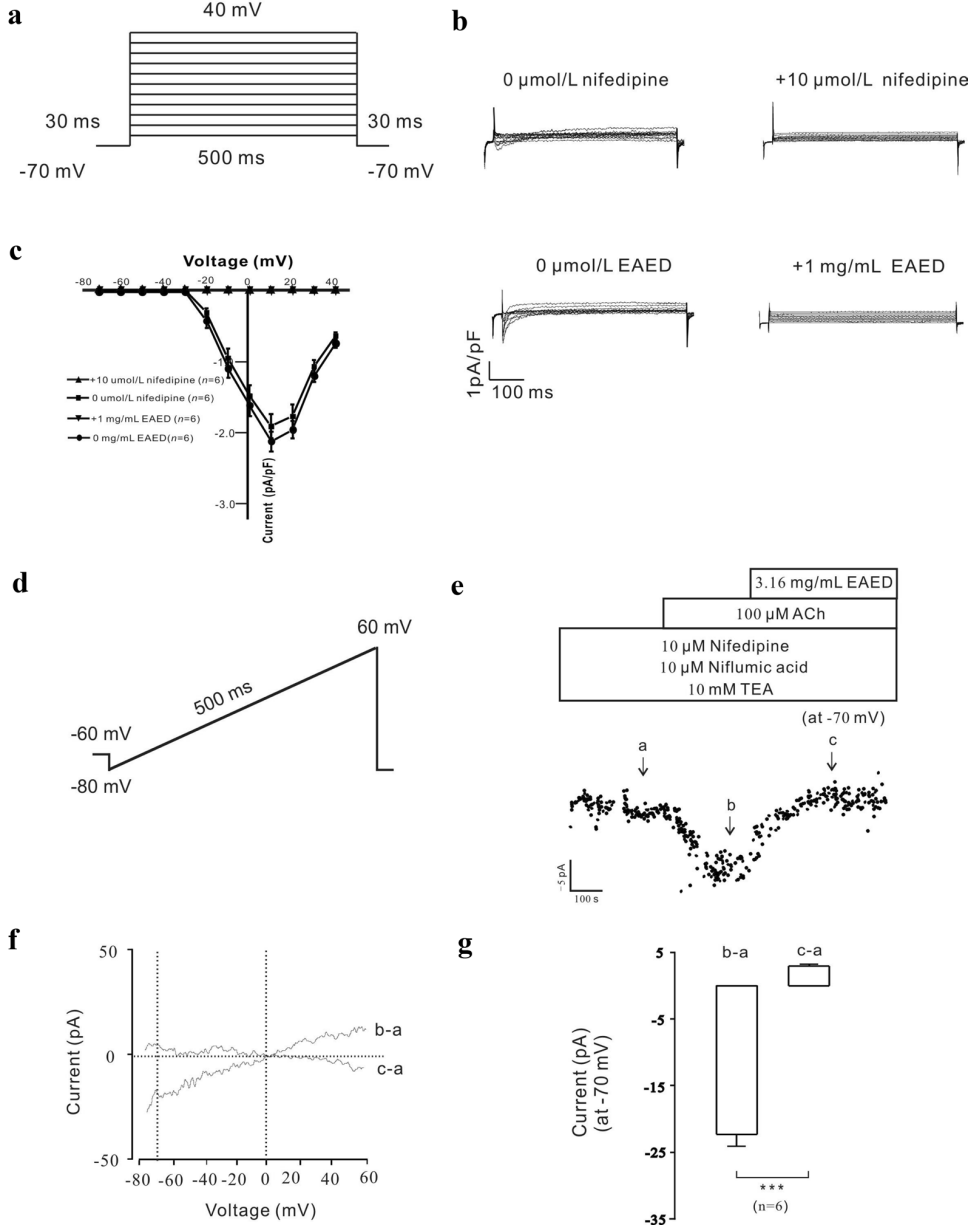
EAED blocks VDLCCs and NSCCs currents

To further clarify the underlying mechanism, the currents regulated by VDLCCs and NSCCs were measured. As shown in Fig. 7a, the VDLCC current was completely blocked by 10 µM nifedipine and 1 mg/mL EAED. The statistical data of 6 cells examined in each of the two experimental groups showed that + 10 mV, 1 mg/mL EAED, and 10 µM nifedipine completely blocked the current.

To test whether EAED affects the opening of NSCCs, nifedipine, niflumic acid, and TEA were added to exclude the influence of VDLCC, K^+^, and Cl^−^ currents, respectively. The results showed that the NSCC current was blocked by 3.16 mg/mL EAED under − 70 mV voltage conditions (Fig. 7b). These results indicated that EAED can completely inhibit the opening of NSCCs induced by ACh.

### The drug toxicity of EAED is very low at the tissue level

**Fig. 8:**
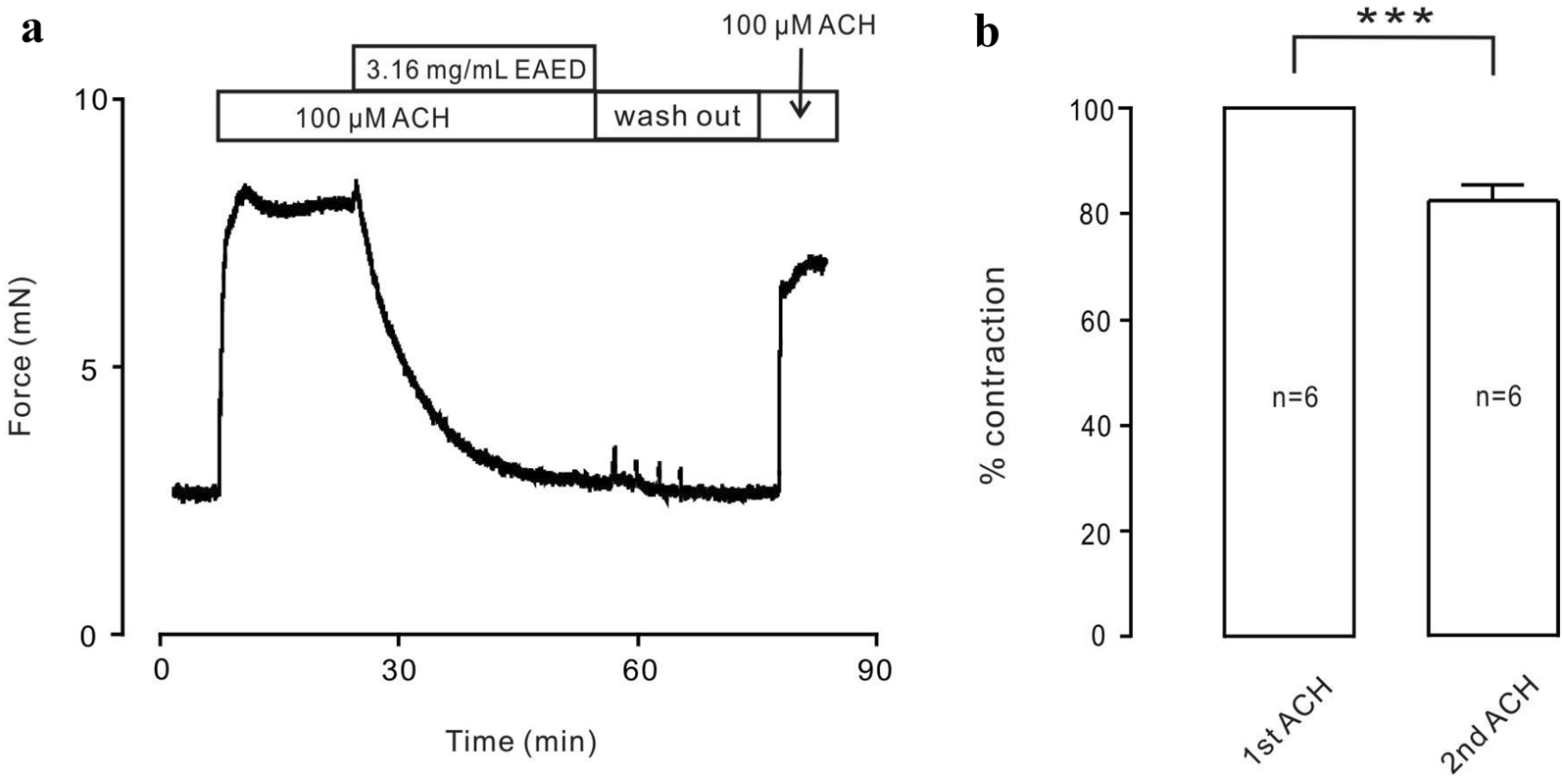
The tracheal rings could still be stimulated to shrink after relaxation by EAED

Next, the toxicity of EAED in mouse TRs was analyzed. After 3.16 mg/mL EAED completely blocked the contraction induced by ACh, the TRs were eluted and balanced for a period of time, again with ACh stimulation, and the contraction apparently occurred again (Fig. 8a). The second ACh-induced shrinkage was about 81% that of the first (Fig. 8b). The above results showed that EAED had little effect on the activity of TRs when relaxing them and could be used in in vivo experiments.

### EAED reduces the respiratory resistance induced by ACh in control and asthma groups

**Fig. 9:**
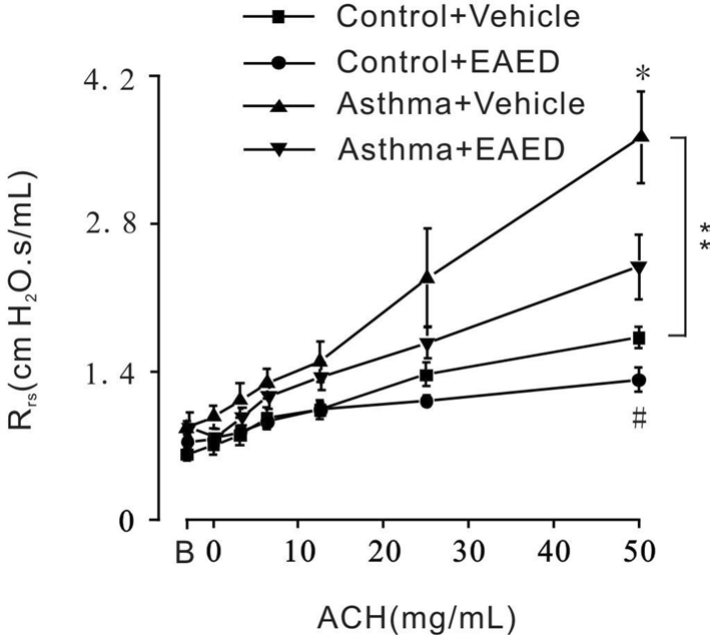
EAED reduced the respiratory resistance induced by ACH in the control group and the asthma group

To investigate whether EAED could potentially improve airway hyperresponsiveness in mice, the lung functions of groups of healthy or asthmatic mice were assessed by the forced oscillation technique at baseline and after exposure to doubling concentrations of aerosolized ACh (3.125–50 mg/mL) dissolved with vehicle or EAED. Under baseline conditions, the four experimental groups studied were indistinguishable with the forced oscillation technique. When the ACh concentration was increased to 25–50 mg/mL, the atomized EAED dissolved with ACh significantly reduced the respiratory resistance of the control and asthma groups compared with the vehicle group (Fig. 9). As expected, the asthmatic mouse group demonstrated ACh-sensitive hyperresponsiveness compared with the control group, particularly after the addition of 25 and/or 50 mg/mL aerosol ACh.

## Discussion

In this study, we found that EAED reduced both high K^+^- and ACh-induced precontractions in mouse TRs and lung slices by inhibiting L-type Ca^2+^ channels and additional TRPC3 and/or STIM/Orai channels, respectively. EAED suppressed the cytoplasmic Ca^2+^ concentration elevation caused by high K^+^ and ACh. In addition, in an in vivo study, EAED effectively reduced the elevated respiratory resistance, *R*_rs_, induced by ACh in healthy and asthmatic mice.

As mentioned above, β2 adrenergic receptor agonists are often used as first-line bronchodilators to relieve asthma [26], but their use has been associated with many adverse effects and high recurrence rates. Thus, we aimed to identify bronchodilators derived from plants. We first extracted a component—EAED—from dandelion. To investigate whether EAED has a diastolic effect on the ASM of mice, two stimuli—high K^+^ and ACh—were applied to elicit mouse TR precontractions and examine the effects of EAED. The experimental results showed that EAED markedly antagonized both high K^+^- and ACh-induced TR contractions in mice, with the maximum relaxant efficiency reaching almost 100% (Figs. 1 and 2). These data demonstrated that EAED possesses relaxant potency against contractions induced by ACh/high K^+^ stimulation.

We further investigated the underlying mechanism of the EAED-mediated relaxant effects on high K^+^-induced contraction. The experiments were conducted under 0 and 2 mM extracellular Ca^2+^ conditions. High K^+^ caused membrane depolarization, leading to the activation of VDLCCs [27]. In our study, high K^+^-induced contractions were completely abolished in Ca^2+^-free medium (Fig. 5a), suggesting that this type of contraction may be dependent on Ca^2+^ influx through VDLCCs. Moreover, EAED completely blocked VDLCC currents (Fig. 7a). These data indicated that EAED relaxed high K^+^-induced contraction by blocking VDLCC-mediated Ca^2+^ influx (Fig. 5a and b).

We then explored the pathways involved in EAED-mediated relaxation of ACh-induced contractions. We found that nifedipine partially inhibited ACh-induced sustained contractions under 2 mM Ca^2+^ conditions but had no effect on ACh-induced transient contractions under 0 Ca^2+^ conditions (Figs. 4a and 5c). These results suggested that VDLCCs were responsible for the long-lasting contractions triggered by extracellular Ca^2+^ influx, but not intracellular Ca^2+^ release-induced transient contractions. However, EAED almost entirely eliminated both of these two types of contractions (Fig. 5d), which suggests that the relaxant effects of EAED depend on blockade of both the extracellular Ca^2+^ influx-mediated by VDLCCs and the intracellular Ca^2+^ release.

Moreover, TRPC3 and/or STIM/Orai channels, as NSCCs, also play roles in ACh-induced contractions by mediating Ca^2+^ influx [28]. Our results indicate that Pyr3 can also cause partial inhibition in the presence of nifedipine, which proves that TRPC3 and/or STIM/Orai channels are also involved in the contraction process. In addition, the NSCC current was effectively blocked by EAED (Fig. 7b). However, in addition to L-type Ca^2+^ channels and NSCCs, other channels may mediate the ACh-induced contractions further inhibited by EAED (Fig. 4c). These unknown mechanisms require additional investigation.

The above experimental results were all obtained using the main trachea of mice. Moreover, our subsequent experiments conducted in lung slices suggested that EAED could also inhibit bronchial smooth muscle contraction (Fig. 3), indicating that EAED is able to entirely block ASM contraction. In terms of Ca^2+^ dynamics in ASMCs, we further showed that EAED decreased high K^+^− and ACh-mediated increases in intracellular Ca^2+^ (Fig. 6). In addition, our in vivo work revealed that EAED could relieve respiratory resistance in healthy and asthmatic mice (Fig. 9).

### Conclusion

In summary, this study demonstrated that EAED inhibits agonist-induced sustained contractions of ASM by inhibiting several types of ion channels, decreases agonist-induced elevation of the cytosolic free Ca^2+^ concentration in ASMCs, and relieves respiratory resistance in healthy and asthmatic mice. Meanwhile, unknown pathways might also be involved in EAED-mediated relaxation, in addition to VDLCCs and NSCCs. These results suggest that EAED could be a new inhibitor of asthma attacks.

## Data Availability

The data and materials supporting the conclusions are included within the article and its supplementary information files.

## Abbreviations

EAED: Ethyl acetate extract from Dandelion
ASM: Airway smooth muscle
ASMCs: Airway smooth muscle cells
FOT: Forced oscillation technique
TMHM: Taraxacum mongolicum Hand.-Mazz.
LPS: Lipopolysaccharide
ACH: Acetylcholine chloride
Pyr3: pyrazole3
SPF: Specific pathogen free
Rrs: Respiratory system resistance
